# Urinary Cells Flow Cytometry in Renal Disease: A Systematic Review of Diagnostic and Prognostic Applications

**DOI:** 10.3390/biomedicines14051050

**Published:** 2026-05-06

**Authors:** Rosa Dolores Prieto-Utrera, Juan Manuel Priede-Vimbela, Marc Vives, Pablo Jorge-Monjas, David Bernardo, Álvaro Tamayo-Velasco, Rodrigo Poves-Álvarez, Eduardo Tamayo, Adrián García-Concejo

**Affiliations:** 1 BioCritic, Group for Biomedical Research in Critical Care Medicine, Ramon y Cajal Ave. 7, 47005 Valladolid, Spain; rosadprieto@gmail.com (R.D.P.-U.); pjorgemo@saludcastillayleon.es (P.J.-M.); alvaro.tamayo@uva.es (Á.T.-V.); rpoves@saludcastillayleon.es (R.P.-Á.); eduardo.tamayo@uva.es (E.T.); adrian.garcia.concejo@uva.es (A.G.-C.); 2 Institute of Health Sciences of Castile and Leon (ICSCYL), 42002 Soria, Spain; 3 Research Unit, University Clinical Hospital of Valladolid, 47003 Valladolid, Spain; 4 Department of Anesthesiology and Critical Care, University Clinical Hospital of Valladolid, 47003 Valladolid, Spain; jmpriede@gmail.com; 5 Department of Anesthesia & Critical Care, Unversity Clinic of Navarra, 31008 Pamplona, Spain; 6 Instituto de Investigación Sanitaria de Navarra (IdiSNA), 31008 Pamplona, Spain; 7 Network Biomedical Research Center for Infectious Diseases (CIBERINFEC), Instituto de Salud Carlos III, 28029 Madrid, Spain; 8 Mucosal Immunology Lab, Unit of Excellence, Institute of Biomedicine and Molecular Genetics of Valladolid (IBGM), University of Valladolid-CSIC, 47003 Valladolid, Spain; d.bernardo.ordiz@gmail.com; 9 Haematology and Hemotherapy Department, University Clinical Hospital of Valladolid, 47003 Valladolid, Spain; 10 Department of Surgery, Faculty of Medicine, University of Valladolid, 47003 Valladolid, Spain

**Keywords:** flow cytometry, urine, urinary cells, biomarkers, markers, diagnosis

## Abstract

**Background:** Urinary cellular biomarkers detected by flow cytometry have emerged as promising non-invasive tools for the diagnosis, prognosis, and monitoring of renal and urological diseases. However, a comprehensive synthesis of their clinical applicability is lacking. **Objectives:** This review aimed to systematically evaluate and summarize the evidence of urinary cellular biomarkers identified through flow cytometry in human populations with renal or urological diseases. **Methods:** A systematic search of PubMed, Scopus and Web of Science was conducted from inception to January 2025. Observational studies analyzing urinary samples by flow cytometry were included, whereas animal studies, genetic-only analyses and studies with incomplete data were excluded. Data extraction and risk of bias assessment were performed independently by two reviewers using a standardized form and the Newcastle-Ottawa Scale. Due to heterogeneity in study populations, designs, and cytometry methods, results were synthesized narratively. **Results:** Of 3938 records screened, 23 studies met the inclusion criteria. All studies applied flow cytometry to characterize urinary cellular biomarkers across renal diseases. Several studies reported promising diagnostic and monitoring applications, but substantial heterogeneity in study design, cytometry protocols, and marker panels limited comparability. Longitudinal analyses and robust prognostic validation were infrequently reported. **Conclusions:** Urinary cellular biomarkers assessed by flow cytometry represent a promising non-invasive approach for evaluating renal and urological diseases. However, clinical implementation remains constrained by heterogeneity and insufficient validation. Future research should focus on standardized methodologies and large prospective studies to establish their diagnostic and prognostic utility.

## 1. Introduction

Flow cytometry is a widely used analytical technique in clinical and biomedical research due to its capability to rapidly and accurately analyze the physical and biological characteristics of cells, including multiparametric analyses of individual cells in suspension and the identification of surface markers [1]. This technology has been applied successfully to biological samples such as blood and urine, demonstrating its potential in the diagnosis and monitoring of various pathological conditions [2].

Currently, the diagnosis and monitoring of patient physiological and immune status to evaluate disease progression is commonly performed using traditional clinical methods, many of which are invasive, time-consuming or resource intensive [3]. In contrast, urinary flow cytometry provides a non-invasive alternative that allows for the rapid evaluation of cellular components excreted in urine, with potential applications in the early detection and monitoring of renal diseases, including urinary tract infection screening and other inflammatory conditions [4]. Urine has also been increasingly recognized as a clinically relevant “liquid biopsy” source for biomarker discovery in both renal and urological diseases [5].

Urinary flow cytometry enables the comprehensive evaluation of biological processes associated with tissue damage and repair, including the recruitment of inflammatory cells and the activation and differentiation of immune cells [6]. These processes are clinically relevant in systemic renal disorders and related immunological conditions where the urinary sediment reflects underlying pathophysiology. Monocyte- and macrophage-driven inflammatory pathways have been strongly implicated in renal injury, and their activity may be reflected in urinary immune cell profiles [7]. Multiple studies have investigated the performance of urinary flow cytometry in detecting and quantifying cells such as bacteria, leukocytes and epithelial cells, comparing its diagnostic accuracy to standard urine culture methods [8]. For example, flow cytometry systems like the Sysmex UF-1000i have demonstrated high sensitivity and specificity in predicting bacteriuria and pyuria and can reduce unnecessary urine cultures in clinical microbiology laboratories [9].

Despite this, limitations remain in the standardization of protocols and data interpretation, which impede the broad clinical implementation of urinary flow cytometry as a routine diagnostic tool [10]. Nevertheless, urinary flow cytometry represents a promising platform for integrating novel cellular biomarkers that may improve early diagnosis and disease stratification, particularly in renal disorders where non-invasive monitoring is desirable [11]. Recent evidence also supports the utility of urinary biomarkers in the non-invasive assessment of acute and chronic kidney diseases, reinforcing their role in disease stratification and monitoring [12]. The incorporation of new urinary biomarkers alongside traditional parameters may enhance early diagnosis and offer deeper insights into disease pathogenesis, particularly in conditions where non-invasive monitoring is advantageous [4].

Therefore, this systematic review aims to identify, evaluate, and synthesize the available evidence on the use of flow cytometry in urinary samples for the diagnosis and monitoring of renal diseases, with emphasis on immune-mediated, inflammatory and transplant-related conditions, as well as selected urological applications. Particular focus is placed on methodological approaches, biomarker characterization and clinical applicability.

## 2. Materials and Methods

This review was conducted through the collection and analysis of existing studies in order to address the uncertainties that prevail in the literature regarding the use of flow cytometry in urine samples. The review was carried out following the guidelines established in the PRISMA 2020 guidelines for systematic review and meta-analysis reporting [13]. The PRISMA 2020 checklist and PRISMA abstract checklist have been completed and are provided in the Supplementary Materials. By adhering to these strict standards, this study contributes to a stronger understanding of the role of flow cytometry in determining urinary biomarkers.

### 2.1. Eligibility Criteria

This systematic review included studies that investigated urinary cellular biomarkers using flow cytometry in human populations with suspected or confirmed renal or urological diseases. The review question was defined based on population (patients with renal diseases), intervention/exposure (urinary flow cytometry) and outcomes (identification and clinical relevance of cellular biomarkers). Eligible studies analyzed urinary samples by flow cytometry and reported the identification, characterization or clinical application of cellular biomarkers. Only observational study designs, cohort, case-control, cross-sectional, longitudinal, prospective or retrospective, were considered. Studies were excluded if they involved animal subjects, focused exclusively on genetic or molecular analyses without flow cytometry application, contained insufficient or incomplete data or were reviews, editorials, conference abstracts or commentaries. For synthesis, studies were grouped according to disease type and by biomarker class.

### 2.2. Information Sources

A comprehensive literature search was conducted in PubMed, Scopus and Web of Science from inception through to January 2025, to ensure a complete and exhaustive retrieval of studies given the emerging nature of urinary flow cytometry-based biomarker research. Reference lists of included studies and relevant reviews were also manually screened to identify additional eligible publications. No language restrictions were applied to maximize the inclusivity of the search.

### 2.3. Search Strategy

The search combined Medical Subject Headings (MeSH) and free-text terms using Boolean operators AND/OR. Key terms included “flow cytometry” or “cytofluorometry” in combination with “biomarkers” or “markers” and “urine”, “urinary cells” or “urinary sediment.” Only studies involving human participants were retained. Full search strategies for each database are provided in Table 1.

The systematic search was conducted using combinations of keywords grouped into three main categories: flow cytometry terms (Flow cytometry OR Cytofluorometry), urine-related terms (Urine OR Urinary cell OR Urinary sediment) and biomarkers (Biomarkers OR Markers). Keywords within each category were combined using OR, and the three categories were combined using AND. Studies involving animal models or focusing exclusively on genetic analyses were excluded. The search was applied across the Web of Science, Scopus and PubMed databases.

### 2.4. Selection Process

Two reviewers independently screened titles, abstracts, and full texts, resolving disagreements through discussion and consensus. Reference management was performed using Mendeley (Elsevier, version v2.144.0) and no automation tools were used. Screening was conducted independently and not blinded to study authors or journals. The study selection process followed PRISMA 2020 guidelines and is detailed in the PRISMA flow diagram.

### 2.5. Data Collection Process

Data was extracted independently by two reviewers using a standardized pre-defined form. Extracted information was cross-checked for accuracy and any disagreements were resolved through discussion. Data was obtained directly from the main text, tables, and Supplementary Materials; no additional contact with study authors was required.

### 2.6. Data Items

Primary outcomes included the type and frequency of urinary cellular biomarkers, such as T cells (CD4, CD8, CD25, Foxp3), B cells (CD19, CD27, CD38), plasmacytoid dendritic cells, monocytes/macrophages (CD14, CD16, CD68, CD11b, CD206, CD163), tubular epithelial cells (CD10) and podocalyxin-positive cells. The clinical relevance of these biomarkers for diagnosis, prognosis, or disease monitoring was also extracted. Additional variables included study characteristics (authors, year, country), study design and sample size, participant demographics (age, sex, disease type), flow cytometry methodologies and funding sources.

### 2.7. Study Risk of Bias Assessment

Methodological quality and risk of bias were assessed using the Newcastle–Ottawa Scale (NOS) for observational studies [14]. This tool evaluates three domains: selection of study groups, comparability of groups, and ascertainment of the outcome or exposure. Each study was awarded up to nine stars and classified as low (7–9 stars), moderate (4–6 stars), or high risk of bias (0–3 stars). Two reviewers independently assessed each study, and disagreements were resolved through discussion and consensus.

### 2.8. Effect Measures

Given the substantial heterogeneity across included studies in terms of study design, patient populations, disease entities, flow cytometry protocols, and outcome reporting, a quantitative meta-analysis was not feasible. Therefore, results are presented using a narrative synthesis approach. Given the narrative nature of this review, no quantitative effect measures were calculated and all results are presented qualitatively.

### 2.9. Synthesis Methods

Studies were grouped by disease type and biomarker class. All reported outcomes were standardized to facilitate comparison, with missing data indicated as “not reported.” Key findings are summarized in Table 2 and Table 3. A narrative synthesis was performed due to heterogeneity in study designs, populations, biomarkers and measurement techniques. Differences across studies were discussed in terms of study design, population characteristics and cytometry methodologies. Sensitivity analyses were not applicable.

### 2.10. Reporting Bias Assessment

No formal assessment of publication bias was performed due to the absence of meta-analysis. The narrative evaluation of potential selective reporting and study limitations are included in the Section 4.

### 2.11. Certainty Assessment

The certainty of evidence for each outcome was qualitatively evaluated, considering risk of bias, inconsistency, indirectness, imprecision and the relevance of study findings.

### 2.12. Patient and Public Involvement

Patients and the public were not involved in this review, as it was based exclusively on previously published data.

This systematic review was prospectively registered in PROSPERO (Registration number: CRD420261355392) and conducted in accordance with the PRISMA 2020 guidelines. A predefined protocol was used to define the review objectives, eligibility criteria, search strategy and data extraction process.

## 3. Results

The results should provide a concise and precise description of the experimental results, their interpretation, as well as the experimental conclusions that can be drawn.

### 3.1. Study Selection

A systematic search was conducted in Web of Science, Scopus, and PubMed, identifying a total of 3938 records (Web of Science: 3354; Scopus: 205; PubMed: 379). After removing duplicates and records deemed irrelevant before screening (*n* = 2574), 1364 records remained for title and abstract screening.

Of these, 1172 records were excluded based on title and abstract. Full-text reports were retrieved for 192 studies, and none were lost at this stage. After eligibility assessment, 169 reports were excluded for the following reasons: incomplete abstracts (*n* = 42), insufficient or non-relevant data (*n* = 95), or study design not observational (*n* = 32).

**Figure 1:**
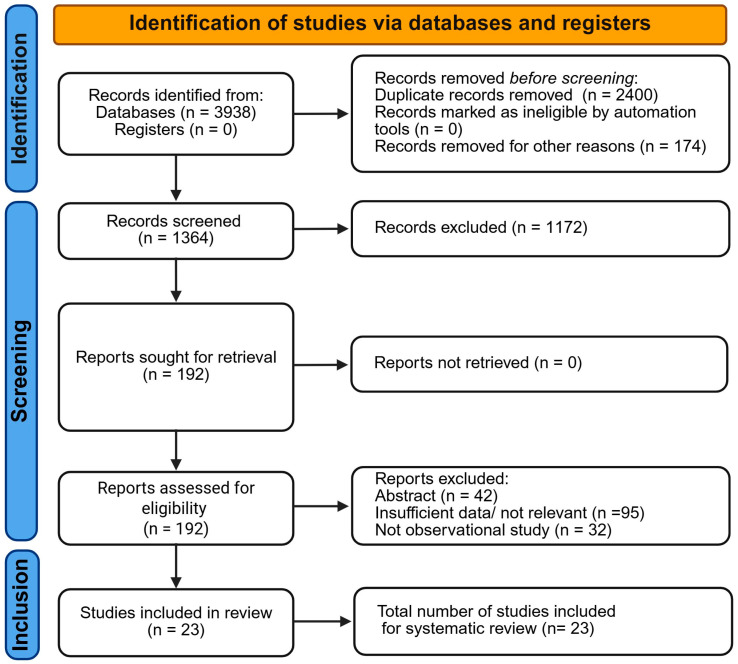
PRISMA 2020 flow diagram of study selection. A total of 3938 records were identified from Web of Science (*n* = 3354), Scopus (*n* = 205), and PubMed (*n* = 379). After removing 2574 duplicates and irrelevant records prior to screening, 1364 records were screened by title and abstract, of which 1172 were excluded. Full-text articles were retrieved for 192 studies, with none lost at this stage. Of these, 169 studies were excluded due to incomplete abstracts (*n* = 42), insufficient or non-relevant data (*n* = 95), or non-observational study design (*n* = 32). Ultimately, 23 studies met all inclusion criteria and were included in the systematic review.

Ultimately, 23 studies met all inclusion criteria and were included in this systematic review. Figure 1 depicts the study selection process in a PRISMA 2020 flow diagram.

### 3.2. Urinary Cellular Populations Identified by Flow Cytometry

Urine contains diverse cellular populations that can be analyzed by flow cytometry, including immune cell subsets (T lymphocytes, B lymphocytes, monocytes/macrophages and dendritic cell populations) as well as epithelial-derived cells. These populations can be phenotypically characterized using combinations of surface and intracellular markers, enabling the identification of disease-associated immune signatures across a range of renal and systemic conditions.

Several studies suggest that urinary cellular profiles may partially reflect intrarenal immune and inflammatory processes observed in kidney biopsy specimens, supporting their potential role as non-invasive indicators of renal immune activity.

Across the included studies, urinary cell populations were defined using multiparametric flow cytometry based on lineage-defining markers (e.g., CD3, CD4, CD8, CD19, CD14), functional and regulatory markers (e.g., CD25, Foxp3), chemokine receptors (e.g., CXCR3, CCR7), and activation or differentiation markers (e.g., CD11c, CD45RO). This approach allows the identification of specific immune cell subsets rather than isolated markers. All included studies are summarized in Table 2.

#### 3.2.1. Autoimmune Diseases

Flow cytometry has proven to be a crucial tool for identifying key cellular populations in the urine of patients with Lupus Nephritis (LN) [37]. Studies have found that T cells subsets (CD4^+^ and CD8^+^) and monocyte/macrophage populations (CD11c^+^ cells) in urine are promising indicators for evaluating renal inflammatory activity and response to treatment in LN patients [15]. An imbalance in the CD4:CD8 ratio has also been observed, which could provide important insights into disease progression [16]. Moreover, CD11c macrophage subsets have shown high predictive value in assessing the renal response to immunosuppressive treatment [21,38].

In Systemic Lupus Erythematosus (SLE) patients, urinary immune cell subsets including regulatory T cells (CD4^+^CD25^+^Foxp3^+^), B cell populations, and plasmacytoid dendritic cells have been associated with disease activity and renal involvement [25,27]. Studies have shown that CXCR3^+^ T cells are enriched in the urine and are used as key biomarkers to monitor renal disease activity in SLE [26].

In ANCA-associated Vasculitis (AAV), urinary CD4 T cell subsets, particularly effector memory cells, are present in the urine of patients and serve as valuable biomarkers for assessing renal involvement. Monitoring these cells can assist in predicting disease progression [24,34].

#### 3.2.2. Transplant-Related Conditions

Flow cytometry has also been used to evaluate immune subsets in the urine of renal transplant patients, providing insights into transplant rejection. Studies highlight that T cells, monocytes and tubular epithelial cells are key indicators for monitoring renal rejection and could facilitate the non-invasive detection of this phenomenon [29].

#### 3.2.3. Metabolic and Other Nephropathies

In other renal conditions such as diabetic nephropathy and idiopathic membranous nephropathy, urinary podocyte injury has been assessed through the detection of podocalyxin-positive cells and has emerged as a potentially potential marker for evaluating and monitoring disease progression [32,33]. Differences in the prevalence of podocalyxin-positive cells have also been observed in obese patients, suggesting their potential use in metabolic studies and renal diseases associated with obesity [31].

In summary, urinary flow cytometry-derived cellular profiles represent a promising non-invasive approach for characterizing renal and systemic inflammatory diseases. However, due to heterogeneity in study design, patient populations, and immunophenotyping panels, further standardized and prospective studies are required to confirm their diagnostic and prognostic utility.

### 3.3. Risk of Bias in Included Studies

The methodological quality of the included studies was assessed using NOS. Overall, most studies were classified as having a moderate risk of bias (Table 4). Specifically, six studies were classified as at low risk of bias (scores ≥ 7), while the remaining studies were considered to have moderate risk. No studies were identified as having a high risk of bias. The main sources of potential bias included limited comparability between study groups, relatively small sample sizes, and the lack of longitudinal follow-up in several studies.

## 4. Discussion

This systematic review demonstrates that urinary flow cytometry enables the identification and characterization of diverse cellular populations in urine, including immune cell subsets (T lymphocytes, B lymphocytes and monocytes/macrophages) as well as epithelial-derived cells. Across the 23 included studies, these populations were associated with different aspects of renal pathology, including inflammation, immune activation and tissue injury. Importantly, the clinical value of urinary flow cytometry should be interpreted in the context of established diagnostic methods, including renal biopsy, urine microscopy and microbiological culture. Overall, these findings highlight the potential of urinary flow cytometry as a non-invasive tool to assess renal inflammation, injury, and repair, providing valuable information that complements conventional biochemical markers.

The efficacy of urinary flow cytometry lies in its ability to perform rapid multiparametric analysis and directly assess antigen expression, enabling not only identification of cellular populations but also their integration into broader pathophysiological patterns. Importantly, the combined interpretation of urinary cellular signatures allows stratification into major biological processes, including inflammatory immune infiltration, epithelial injury, immune-mediated tissue damage and repair/regenerative responses [39]. Inflammatory immune infiltration, characterized by the presence of CD4^+^ and CD8^+^ T-cell subsets together with CD14^+^ or CD11c^+^ monocyte/macrophage populations, likely reflects active intrarenal immune infiltration and inflammatory amplification in conditions such as lupus nephritis and SLE [40,41]. Chemokine-driven immune recruitment and activation, illustrated by the enrichment of CXCR3^+^ T cells, suggests the targeted migration of effector lymphocytes into inflamed renal tissue [42,43]. Immune-mediated tissue injury and structural damage is particularly evident in transplant settings, where the combined detection of lymphoid, myeloid, and epithelial-derived cells reflects both immune activation and parenchymal injury [29]. Epithelial injury and cellular exfoliation, associated with the presence of tubular epithelial cells and injury-related markers, is indicative of active tissue damage and repair and regenerative processes suggested by emerging cellular populations such as urine-derived stem cells, which may contribute to renal recovery mechanisms. These cellular profiles reflect underlying immunopathological mechanisms, including inflammation, immune cell recruitment and tissue repair, which may explain the ability of flow cytometry to detect early changes not captured by conventional biochemical markers.

While several studies report the presence of tubular epithelial cells in urine, fewer have explored their functional or injury-related phenotypes. Exfoliated renal epithelial cells may express markers associated with cellular stress and injury, such as kidney injury molecule-1 (KIM-1) and neutrophil gelatinase-associated lipocalin (NGAL), which reflect active tubular damage rather than simple cell shedding [44,45]. The incorporation of such markers into flow cytometry panels could enhance the biological and clinical interpretation of urinary cellular analysis, particularly in acute kidney injury and transplant rejection [46]. However, current evidence remains limited, and most studies focus primarily on cellular identification rather than functional characterization. An additional limitation of the current literature is the underrepresentation of non-immune and regenerative cell populations. Urine-derived stem cells (USCs), which exhibit mesenchymal stem cell-like properties, have emerged as a potential component of renal repair and regeneration processes [47]. Beyond their biological relevance, USCs have been proposed as a non-invasive source of patient-specific cells with potential applications in disease modeling, biomarker discovery and regenerative medicine. However, their characterization by flow cytometry in urinary samples remains scarce, highlighting an important gap in the current evidence base.

Urinary flow cytometry should be interpreted within the context of established diagnostic methods, including renal biopsy, urine microscopy and microbiological culture. Renal biopsy remains the gold standard for histopathological evaluation, providing detailed structural and cellular information; however, its invasive nature limits its use for repeated assessments [48]. In contrast, urinary flow cytometry offers a non-invasive and repeatable approach with the potential to reflect intrarenal immune and inflammatory processes in real time [49]. Compared with conventional urine microscopy, which provides limited morphological information and urine culture, which primarily detects infectious agents without characterizing host immune responses, flow cytometry enables the detailed immunophenotypic analysis of urinary cellular populations [50].

In this context, urine represents an accessible and underutilized biological fluid for the non-invasive study of renal epithelial and immune cells in a wide range of kidney diseases, including diabetic nephropathy and focal segmental glomerulosclerosis [51]. The application of urinary flow cytometry may therefore reduce the need for invasive procedures and shorten time to diagnosis. Additionally, the rapid reporting of negative results could help minimize unnecessary empirical treatments [29]. The technique is feasible in most laboratory settings and, when combined with morphological evaluation, can provide a sufficiently accurate differential diagnosis.

Furthermore, urinary biomarkers identified through flow cytometry may enable early detection of renal injury before conventional markers, such as serum creatinine rise, potentially improving patient selection and stratification in clinical trials [6,52]. From a clinical perspective, urinary flow cytometry could be incorporated as a complementary tool for screening, risk stratification, and monitoring of patients with suspected or established renal disease. Its ability to identify cellular profiles associated with inflammatory activity or early rejection may facilitate more precise and timely therapeutic decisions [29,53]. Moreover, the non-invasive nature and accessibility of urine sampling allow for dynamic, repeated assessments, supporting the optimization of individualized therapeutic strategies [54].

Despite these promising findings, several limitations must be considered. Most included studies were observational with small sample sizes, which limit statistical power and increases the risk of bias. In addition, substantial heterogeneity exists across studies in terms of patient populations, disease definitions, and outcome measures. A key limitation is the lack of standardization in flow cytometry protocols, including differences in sample processing, antibody panels, and gating strategies, which reduces reproducibility and comparability. Furthermore, many biomarkers have not been validated against histological findings, and longitudinal studies remain scarce. At the biomarker level, specificity is also a concern. For example, CD10 is not fully specific for tubular epithelial cells and may be expressed by other cell types under pathological conditions, which may lead to misclassification in heterogeneous urinary samples. Overall, these limitations highlight the need for standardized multiparametric approaches and larger prospective studies to validate the clinical utility of urinary flow cytometry and define its diagnostic, prognostic, and monitoring applications.

## 5. Conclusions

Urinary flow cytometry represents a promising non-invasive approach for the evaluation of renal pathology, with potential applications in diagnosis, prognosis, and disease monitoring. Current evidence suggests clinical utility; however, its implementation in routine practice remains limited by methodological heterogeneity and insufficient validation across studies. Future research should focus on standardized protocols, larger prospective cohorts, and systematic correlation with histopathological findings to better define its clinical role. In combination with established diagnostic strategies, urinary flow cytometry may ultimately contribute to earlier detection, improved risk stratification and more personalized management of renal diseases.

## Figures and Tables

**Table 1:** Search strategy for study identification.

Category	Search Terms
Flow Cytometry-related conditions	Flow cytometry OR Cytofluorometry
Urine-related conditions	Urine OR Urinary cell OR Urinary Sediment
Biomarkers-related conditions	Biomarkers OR Markers
Exclusion	No animal OR Genetic studies

**Table 2:** Characteristics of studies included in the systematic review.

Study ID	Country	Study Design	Sample Size	Clinical Condition	Immune Cell Populations and Defining Markers
[15]	Germany	Study cohort	94	LN	T cell (CD4 and CD8), Macrophages (CD14)
[16]	Egypt	Longitudinal case-control study	80	LN, SLE	T cell (CD4 and CD8), Macrophages (CD14)
[17]	Germany	Retrospective cohort observational study	123	LN, ANCA-AAV	T and B cell (CD4, CD19) Macrophages (CD14)
[18]	Germany	Retrospective cohort observational study	147	LN	T cells (CD4)
[19]	Germany	Observational study	46	LN	T cell (CD8)
[20]	Brazil	Study cohort	17	LN	T cell (CD4)
[21]	Republic of Korea	Cohort observational study	61	LN	Macrophages (CD11c)
[21]	Republic of Korea	Cohort observational study	40	LN	Macrophages (CD11c)
[22]	Japan	Observational study	224	GN	Monocytes (CD16)
[23]	The Netherlands	Prospective cohort study	85	GPA	B cell (CD27, CD38)
[24]	The Netherlands	Cohort observational study	51	AAV	Effector memory T (CD4)
[25]	Austria	Cohort observational study	97	SLE	T cell (CD4, CD25, Foxp3)
[26]	Germany	Cohort observational study	38	SLE	T cell (CXCR3)
[27]	USA	Cohort observational study	69	SLE	B cells, T cells and Plasmacytoid dendritic cells
[28]	USA	Prospective cohort observational study	17	Acute/chronic renal allograft rejection	HLA-DR, CD14, CD54
[29]	Germany	Prospective cohort observational study	63	Renal transplant	T cell (CD4, CD8) Monocytes/Macrophages Tubular epithelial cells (CD10) Podocalyxin
[30]	Spain	Cohort observational study	52	SLE	Podocalyxin
[31]	Thailand	Non-randomized cross-sectional study	61	Obesity-related glomerulopathy	Podocalyxin
[32]	Italy	retrospective cohort study	27	IMN	Podocalyxin
[33]	Shandong	Cross-sectional cohort study	96	Diabetic nephropathy	Podocalyxin
[34]	The Netherlands	Cohort observational study	36	ANCA-AAV	T cells (CD4) and effector memory T cells (CD4, CD45RO, CCR7)
[35]	Portugal	Prospective study	28	AKI and sepsis	Leukocytes (CD45), T cells (CD161, CD4, CD127, CD25, CD15, CD1c, CD8) B cells (CD3, CD19) Macrophages (CD11b, CD68, CD206, CD163)

Summary of all studies reporting urinary biomarkers analyzed by flow cytometry. Columns indicate study ID (reference number), country, study design, sample size, clinical condition, and biomarkers measured. Abbreviations: LN, Lupus Nephritis; SLE, Systemic Lupus Erythematosus; AAV, ANCA-associated Vasculitis; IMN, Idiopathic Membranous Nephropathy; GN, Glomerulonephritis; AKI, Acute Kidney Injury.

**Table 3:** Summary of urinary cell populations (phenotype-based) identified by flow cytometry for each clinical condition.

Condition	Cell Populations (Phenotype-Based)	Study Design	Sample Size (*n*)	References
LN	CD4, CD8, CD14	Retrospective Cohort	147	[18]
	CD4, CD8, CD14	Longitudinal Case-Control	80	[16]
	CD4, CD19, CD14	Retrospective Cohort	123	[17]
	CD4	Retrospective Cohort	147	[18]
	CD8	Observational	46	[19]
	CD4	Cohort	17	[20]
SLE	CD4, CD25, Foxp3	Cohort Observational	97	[25]
	CXCR3	Cohort Observational	38	[26]
	T cells, B cells, Plasmacytoid Dendritic Cells	Cohort Observational	69	[27]
ANCA-AAV	CD4	Cohort Observational	51	[24]
	CD4, CD45RO, CCR7	Cohort Observational	36	[34]
GPA	CD27, CD38	Prospective Cohort	85	[23]
GN	CD16	Observational	224	[22]
Kidney Transplant	CD4, CD8, CD14, CD10, Podocalyxin	Prospective Cohort	63	[29]
Acute Renal Rejection	HLA-DR, CD14, CD54	Prospective Cohort	17	[28]
Diabetic Nephropathy	Podocalyxin	Cross-sectional Cohort	96	[33]
IMN	Podocalyxin	Retrospective Cohort	27	[32]
Obesity-Related Glomerulopathy	Podocalyxin	Non-randomized Cross-sectional	61	[31]
Bladder Cancer	CD15, CD66b, CD14, CD16, CD56	Prospective	44	[36]
AKI	CD45, CD161, CD4, CD127, CD25, CD15, CD1c, CD8, CD3, CD11b, CD68, CD206, CD163	Prospective	28	[35]

This table lists the biomarkers analyzed, study design, sample size, and references for all studies included in this systematic review. Abbreviations: LN, Lupus Nephritis; SLE, Systemic Lupus Erythematosus; ANCA, Anti-Neutrophil Cytoplasmic Antibody; AAV, ANCA-Associated Vasculitis; GN, Glomerulonephritis; GPA, Granulomatosis with Polyangiitis; IMN, Idiopathic Membranous Nephropathy; AKI, Acute Kidney Injury; CXCR3, CXC Chemokine Receptor 3; CCR7, CC Chemokine Receptor 7; HLA-DR, Human Leukocyte Antigen-DR isotype; Foxp3, Forkhead Box P3.

**Table 4:** Risk of bias assessment of included studies using the Newcastle–Ottawa Scale (NOS).

Study	Selection	Comparability	Outcome	Total	Risk
[32]	★★★★	★	★★	7	Low
[31]	★★★	★★	★★	7	Low
[15]	★★★	★★	★★	7	Low
[35]	★★★	★	★★	6	Moderate
[17]	★★★	★	★★	6	Moderate
[18]	★★	★	★★	5	Moderate
[19]	★★★	★	★★	6	Moderate
[21]	★★★	★	★★	6	Moderate
[23]	★★★	★	★★	6	Moderate
[25]	★★★★	★	★★	7	Low
[23]	★★★★	★★	★★	8	Low
[19]	★★★	★	★★	6	Moderate
[25]	★★★	★	★★	6	Moderate
[26]	★★★	★	★★	6	Moderate
[22]	★★	★	★★	5	Moderate
[28]	★★★	★	★★	6	Moderate
[33]	★★	★	★★	5	Moderate
[29]	★★	★	★★	5	Moderate
[31]	★★★	★	★★	6	Moderate
[26]	★★★	★	★★	6	Moderate
[33]	★★★	★	★★	6	Moderate

The NOS evaluates three domains: Selection (maximum 4 stars), comparability (maximum 2 stars), and outcome/exposure (maximum 3 stars), with a total score ranging from 0 to 9 stars. Studies were classified as low (5–7 stars), moderate (4–6 stars), or high risk of bias (0–3 stars).

## Data Availability

No new data was created or analyzed in this study. Data sharing is not applicable to this article.

## Supplementary Materials

The following supporting information can be downloaded at: https://www.mdpi.com/article/10.3390/biomedicines14051050/s1, PRISMA 2020 for Abstracts Checklist and PRISMA 2020 Checklist.

## Abbreviations

The following abbreviations are used in this manuscript:

AAV	ANCA-associated vasculitis
AKI	Acute kidney injury
ANCA	Anti-neutrophil cytoplasmic antibody
CCR7	CC chemokine receptor 7
CD	Cluster of differentiation
CXCR3	CXC chemokine receptor 3
Foxp3	Forkhead box P3
GN	Glomerulonephritis
GPA	Granulomatosis with polyangiitis
HLA-DR	Human leukocyte antigen–DR isotype
IMN	Idiopathic membranous nephropathy
LN	Lupus nephritis
NOS	Newcastle–Ottawa Scale
PRISMA	Preferred Reporting Items for Systematic Reviews and Meta-Analyses
SLE	Systemic lupus erythematosus
